# Bioorthogonal Chemistry in Neurobiology: From Imaging to Proteomics

**DOI:** 10.1002/cbic.202500949

**Published:** 2026-10-14

**Authors:** Neil Taylor, João de Sousa‐Lourenço, Ivana Nikić‐Spiegel

**Affiliations:** ^1^ Werner Reichardt Centre for Integrative Neuroscience University of Tübingen Tübingen Germany; ^2^ Luxembourg Centre for Systems Biomedicine (LCSB) University of Luxembourg Esch‐sur‐Alzette Luxembourg

**Keywords:** click chemistry, fluorescence microscopy, neural proteomics, protein labeling, unnatural amino acids

## Abstract

Bioorthogonal click chemistry has emerged as a versatile approach for probing molecules with high selectivity and minimal perturbation. While applicable to diverse biomolecules, its use in protein studies has proven particularly powerful, especially in neurobiology, where precise molecular tools are essential. A key technology enabling such precision is genetic code expansion (GCE), which allows the incorporation of unnatural amino acids (UAAs) bearing bioorthogonal handles into proteins. These UAAs can be introduced site‐specifically to label defined proteins or proteome‐wide for systems‐level analysis. Complementing this, proteome‐wide labeling can be achieved through metabolic incorporation of amino acid analogs. Coupled with copper‐catalyzed or strain‐promoted click reactions, these labeling technologies enable applications ranging from protein visualization to mass spectrometry‐based proteomics. Ongoing advances in UAA incorporation strategies and the chemical diversity of available UAAs continue to broaden the opportunities to study protein expression, localization, and dynamics. In this review, we highlight recent methodological developments in click chemistry‐based protein labeling and their applications in neurobiology.

## Graphical abstract

This review highlights emerging strategies for labeling neuronal proteins, enabling visualization and quantitative analysis of their expression, localization, and dynamics. By combining genetic code expansion (GCE), metabolic labeling, and click chemistry‐based labeling, these approaches provide powerful routes to study proteins in specific cells and subcellular compartments using fluorescence imaging or mass spectrometry analysis.

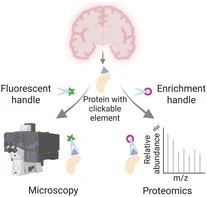

## 1 Introduction

Bioorthogonal click chemistry encompasses a group of chemical reactions that proceed under physiological conditions without perturbing native biological processes. Reactions meeting strict criteria of bioorthogonality must combine high chemical selectivity with biological compatibility, ensuring that neither the functional groups involved nor any required reagents disrupt cellular viability or physiology. Owing to these properties, bioorthogonal strategies have become powerful tools for interrogating the structure, localization, and dynamics of proteins and other biomolecules in living systems, with applications spanning molecular labeling, targeted delivery, and activity‐based profiling [1, 2].

The modern field of bioorthogonal chemistry was strongly shaped by the development of the copper‐catalyzed azide–alkyne cycloaddition (CuAAC, Figure 1i), independently developed by Sharpless and Meldal [3, 4]. Earlier studies of azide reactivity, most notably the metal‐free Staudinger reaction between azides and phosphine, had already highlighted the chemoselectivity and biocompatibility of azides. However, the development of CuAAC represented a major advance in click chemistry by transforming the azide–alkyne cycloaddition into a fast, highly regioselective, and broadly applicable ligation. The reaction proceeds with high yields and rapid kinetics, producing a single 1,4‐disubstituted 1,2,3‐triazole product. However, the requirement for Cu(I) catalysis limits the application of CuAAC in living systems due to copper‐associated cytotoxicity, motivating the development of catalyst‐free alternatives that are more compatible with cellular and in vivo environments.

**FIGURE 1:**
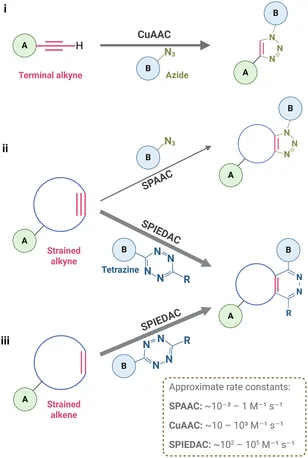
Simplified schematic overview of selected click chemistry reactions. Roman numerals (i–iii) denote the type of reactive functional group shown on molecule A. Molecules A and B represent generic reaction partners (e.g. biomolecules, fluorophores, or affinity/pull‐down reagents), and the complementary reactive groups can be assigned interchangeably to either partner. i) A terminal alkyne on molecule A reacts with an azide‐functionalised molecule B via copper(I)‐catalysed azide–alkyne cycloaddition (CuAAC), in which Cu(I) promotes a 1,3‐dipolar cycloaddition leading to triazole formation. ii) A strained alkyne on molecule A can react, without the need for a catalyst, with either an azide‐functionalised molecule B or a tetrazine‐functionalised molecule B via strain‐promoted azide–alkyne cycloaddition (SPAAC) or strain‐promoted inverse electron‐demand Diels–Alder cycloaddition (SPIEDAC), respectively. iii) A strained alkene on molecule A reacts with a tetrazine‐functionalised molecule B via SPIEDAC. Although tetrazine ligations with strained alkynes (ii) and strained alkenes (iii) both proceed via SPIEDAC, they yield structurally distinct pyridazine‐core adducts. For clarity, the reaction schemes are presented here in a simplified form. Arrow thickness qualitatively reflects relative reaction kinetics under typical biological labelling conditions. Approximate second‐order rate constant ranges are indicated in the lower‐right panel.

Pioneering work by Bertozzi and coworkers established that selective chemical reactions could be designed to proceed within living organisms without copper catalysts, thereby defining the concept of bioorthogonal chemistry and leading to the development of catalyst‐free ligation strategies such as the Staudinger ligation [5] and strain‐promoted azide–alkyne cycloaddition (SPAAC, Figure 1ii) [6]. Subsequent advances across the field introduced additional ultrarapid bioorthogonal reactions, most prominently the tetrazine‐based strain‐promoted inverse electron‐demand Diels–Alder cycloaddition (SPIEDAC) [7, 8] between strained alkenes or alkynes and tetrazines (Figure 1ii,iii). Together, thanks to their high selectivity, compatibility with living systems, and advantageous kinetics, SPAAC and particularly SPIEDAC helped establish the chemical foundation for modern in vivo bioorthogonal labeling and transform click chemistry from an in vitro synthetic method into a broadly applicable strategy for biological studies.

In biological contexts, bioorthogonal labeling is typically achieved through a two‐step procedure. First, small, non‐natural chemical handles are introduced into biomolecules, most commonly by the metabolic incorporation of modified precursors and, in the case of proteins, also by genetic approaches. These handles are then selectively conjugated to reporter probes via a click chemistry reaction. This two‐step workflow has proven especially powerful for the study of proteins, enabling minimally invasive labeling of single proteins as well as proteome‐wide enrichment. Consequently, bioorthogonal chemistry has emerged as a versatile toolkit for probing dynamic protein processes in living systems, with major impact in fluorescence microscopy and proteomics.

Beyond proteins, both copper‐catalyzed and copper‐free click reactions have been successfully applied to other biomolecular classes in the field of neurobiology. Initial studies showed applications of copper‐catalyzed click chemistry to detect proliferating cells in cultured cells and mouse brain tissue through metabolic incorporation of alkyne‐modified nucleosides, such as 5‐ethynyl‐2′‐deoxyuridine (EdU) [9]. A more recent work developed an improved whole‐brain 3D click chemistry workflow combining EdU labeling, tissue clearing, and spatial analysis of neuronal progenitor cells in the mouse brain [10]. The strategy was also applied to RNA labeling, where metabolic incorporation of 5‐ethynyluridine (EU) enabled copper‐catalyzed visualization of newly synthesized RNA in hippocampal neurons and zebrafish brain, showing time‐ and concentration‐dependent labeling, dendritic localization of nascent RNA, and activity‐dependent increases in transcription in neurogenesis‐related regions [11]. More recently, in vivo metabolic RNA labeling in the mouse nervous system combining EU injections with click chemistry was used to visualize newly transcribed RNA in cerebellar neurons and glia [12]. The authors found robust, cell‐type‐specific labeling in neurons (highest in Purkinje cells) and could detect reduced transcription in Purkinje cells of mouse models of neurodegenerative disease. However, high incorporation of EU itself led to Purkinje cell degeneration, suggesting that while short labeling intervals reveal transcriptional changes, EU can be neurotoxic with prolonged exposure.

In addition, metabolic labeling has been applied to neuronal glycans, allowing the visualization of polysialic acid dynamics during hippocampal neuron development using copper‐catalyzed click chemistry [13], as well as imaging of developmental changes in neuronal surface glycosylation in brain tissue cultures through copper‐free approaches [14]. To enable metabolic labeling in the intact brain, the liposome‐assisted bioorthogonal reporter (LABOR) strategy was developed based on the premise that liposomes encapsulating azido sugars could cross the blood–brain barrier [15]. This approach enabled in vivo labeling of newly synthesized sialoglycans in the mouse brain, followed by detection through bioorthogonal click chemistry, providing a method to visualize glycosylation dynamics in living neural tissue. More recently, Xia et al. introduced a fast metabolic glycan labeling (fMGL) strategy using protected ManNAz‐6‐phosphate analogs to enable rapid (≤12 h) click chemistry imaging of sialoglycans in living acute brain slices from mice and human patients [16]. The approach bypasses a rate‐limiting biosynthetic step, producing strong, low‐background labeling in mouse and human brain tissue. Applied to ganglioglioma samples, fMGL revealed elevated sialylation specifically in astrocyte‐like tumor cells, highlighting its utility for rapid analysis of clinical specimens.

Beyond metabolic incorporation strategies, chemoenzymatic approaches have also been developed to interrogate glycosylation in the brain. Hsieh‐Wilson and coworkers developed a two‐step enzymatic labeling platform to study the post‐translational modification O‐GlcNAc. Using an engineered galactosyltransferase to append an azide‐modified galactose selectively onto O‐GlcNAc residues, followed by click chemistry conjugation to biotin, this strategy enables enrichment and identification of O‐GlcNAcylated proteins from the rat brain [17]. The recent extension of this technology to whole‐proteome analyses in mouse liver and brain has enabled the systems‐level mapping of functional O‐GlcNAc networks [18].

Alongside nucleic acids and glycans, lipids are also amenable to click chemistry‐based labeling. For example, one study demonstrated multiplexed bioorthogonal imaging of DNA, RNA, protein, and lipid metabolism in organotypic hippocampal slices, highlighting the versatility of metabolic labeling strategies in complex neural tissue [19]. Furthermore, clickable ω‐alkynyl fatty acid analogs have been used to metabolically label primary hippocampal neurons, enabling detection of protein S‐acylation (palmitoylation) via copper‐catalyzed click chemistry. This approach allows fluorescence imaging and proteomic enrichment of lipid‐modified proteins, providing insight into activity‐dependent lipid modifications that regulate synaptic protein localization and neuronal signaling [20].

In addition to metabolic labeling of lipids themselves, click chemistry–enabled chemical probes are also used to study lipid signaling in the nervous system [21]. For example, selective inhibitors of diacylglycerol lipase α and β (DAGLα/β), key enzymes in endocannabinoid biosynthesis, were validated using activity‐based probes bearing alkyne handles. Following enzyme labeling, CuAAC was used to attach reporter tags for visualization and proteomic analysis. This approach enabled selective target engagement profiling in the brain tissue and revealed that acute DAGL inhibition reshapes lipid signaling networks and affects synaptic function in vivo.

Another exciting application of click chemistry in neurobiology is a ligand‐directed bioorthogonal labeling approach that enables selective, nongenetic visualization and tracking of endogenous neurotransmitter receptors in the living mouse brain. In a recent study, Nonaka and colleagues demonstrated that ligand‐directed bioorthogonal chemistry can selectively label endogenous neurotransmitter receptors in living mouse brains by using alkoxyacylimidazole (LDAI) reagents conjugated to receptor‐specific ligands that tether synthetic probes to target proteins in situ [22]. They showed that these reagents diffuse broadly throughout the brain and covalently modify multiple receptor types (including AMPAR, NMDAR, mGlu1, and GABA_a_R), enabling three‐dimensional mapping of receptor distributions, multicolor imaging, and pulse–chase analysis of receptor dynamics during postnatal development. This work establishes a chemical strategy for tracking the spatial distribution and turnover of endogenous synaptic proteins in intact neural tissue, providing a new tool for in vivo neurobiological studies with high spatiotemporal resolution.

Collectively, these studies demonstrate the breadth of bioorthogonal chemistry for investigating diverse molecular pathways in the nervous system. The aim of this review is to summarize recent advances in the application of bioorthogonal click chemistry to neurobiological research, with particular emphasis on the use of click chemistry for protein labeling in fluorescence microscopy and proteomic studies.

## 2 Incorporation of Bioorthogonal Handles into Proteins

Genetic code expansion (GCE) has emerged as a powerful approach for introducing bioorthogonal handles into proteins [23]. Using GCE, unnatural amino acids (UAAs, also referred to as noncanonical amino acids), which have chemically tailored side chains, can be introduced site‐selectively in vitro and in vivo into target proteins. This is enabled by an orthogonal aminoacyl‐tRNA synthetase (aaRS)/tRNA pair that specifically recognizes a given UAA, together with a unique codon decoded by the orthogonal tRNA during translation (Figure 2A). Because stop codons do not normally encode any canonical amino acids, the orthogonal tRNA is typically engineered to recognize one of them. As a result, a UAA can be incorporated site‐selectively into a protein when the corresponding stop codon is introduced at the desired position in the gene. The amber stop codon is most commonly used among the three stop codons in GCE, and the strategy is therefore often referred to as amber codon suppression.

**FIGURE 2:**
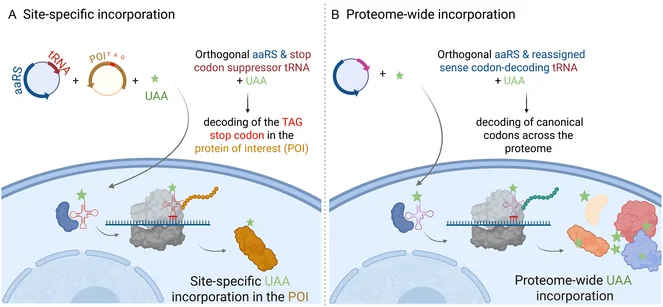
A schematic depiction of using genetic code expansion for site‐selective and proteome‐wide protein labeling in mammalian cells. (A) Site‐selective UAA incorporation using amber stop codon suppression. Plasmids containing genes that encode an orthogonal aminoacyl tRNA synthetase (aaRS)/tRNA pair and the protein of interest (POI) with an in‐frame TAG stop codon are transfected into host cells. The UAA is incorporated into this POI in response to the TAG codon. (B) Proteome‐wide UAA incorporation as in stochastic orthogonal recoding of translation (SORT) labeling using orthogonal aaRS/tRNA pairs. The orthogonal tRNA recognizing the codon of a canonical amino acid is charged with the UAA by the aaRS. The UAA is subsequently stochastically incorporated across the whole proteome in place of the canonical amino acid.

In mammalian cells, pyrrolysine (Pyl)‐specific amber codon suppressor tRNA^Pyl^ and its cognate aaRS (PylRS) from *Methanosarcina* species (*M. barkeri* and *M*. mazei) are the most widely used orthogonal aaRS/tRNA pair for GCE‐based protein labeling. Among the UAAs incorporated using different engineered PylRS variants, UAAs containing azide and tetrazine motifs [24, 25], as well as the most commonly used strained alkyne and alkene derivatives of L‐lysine, such as cyclooctyne (SCO), bicyclononyne (BCN), cyclopropene (CP), and trans‐cyclooctene (TCO) derivatives (Figure 3) have been successfully applied in SPAAC and SPIEDAC‐based protein labeling in different biological systems from prokaryotes to eukaryotes [26, 27, 28, 29, 30].

**FIGURE 3:**
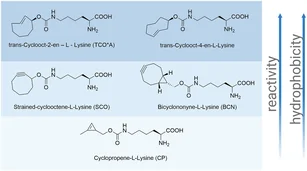
The structures of selected widely used L‐lysine–based UAAs bearing strained alkenes or alkynes for SPIEDAC or SPAAC reactions in microscopy and proteomics.

In addition to site‐specific protein labeling using stop‐codon suppression, some studies have employed orthogonal tRNAs that recognize codons for canonical amino acids [31]. In this strategy, the orthogonal tRNA competes with the endogenous tRNA pool, resulting in stochastic incorporation of UAA throughout the proteome (Figure 2B). This approach, termed stochastic orthogonal recoding of translation (SORT), is described in detail in the sections below. Although SORT sacrifices site‐specific precision, it enables proteome‐wide incorporation of UAAs that can subsequently be functionalized via bioorthogonal click chemistry, which enables enrichment strategies for proteomic analyses or fluorescent dye labeling for visualization by microscopy.

Complementary metabolic labeling strategies rely on amino acid analogs that are processed by the endogenous translational machinery, where the analog competes with the canonical amino acid for activation by the native synthetase and is subsequently incorporated via the cognate endogenous tRNA. Depending on the downstream application, this strategy is referred to as bioorthogonal noncanonical amino acid tagging (BONCAT) or fluorescent noncanonical amino acid tagging (FUNCAT) (Figure 4), with labeled proteins either enriched using biotin (BONCAT) or visualized by fluorescent tagging (FUNCAT).

**FIGURE 4:**
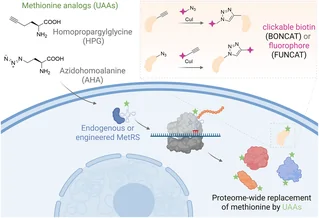
BONCAT and FUNCAT: the UAA homopropargylglycine (HPG) or azidohomoalanine (AHA) is added to the host cell, where it is recognized by the endogenous machinery for the incorporation of methionine. The UAA is therefore incorporated across the proteome in place of methionine. A downstream CuAAC click reaction with an azide‐ or alkyne‐containing compound functionalizes the proteins with a fluorophore (FUNCAT) or biotin handle (BONCAT) for downstream analysis.

In addition to methods relying on amino acid substitution during translation, an alternative strategy employs clickable analogs of puromycin, most commonly O‐propargyl‐puromycin (OPP), for clickable puromycin‐based nascent protein labeling [32]. OPP is incorporated into nascent polypeptide chains as a chain‐terminating aminoacyl‐tRNA mimic, thereby enabling selective bioorthogonal tagging and detection of newly synthesized proteins (Figure 5).

**FIGURE 5:**
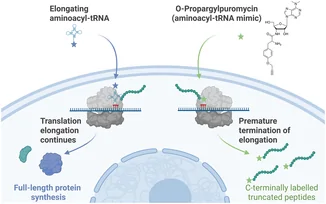
Labeling of nascent peptides using functionalized puromycin. The structure of puromycin resembles that of the 3' end of a tRNA charged with a tyrosine residue. When added to the host cell, a puromycin derivative functionalized with a clickable handle will bind to the ribosome in place of the aminoacyl‐tRNA and be incorporated at the C‐terminus of the peptide being synthesized. This terminates translation, and the C‐terminally labeled peptides can be identified by downstream click reaction with various handles.

Over the last decade, the rapid expansion of GCE methodologies has led to substantial technical advances, many of which have been reviewed extensively [33, 34]. Together with parallel developments in metabolic labeling using reactive amino acid analogs, these approaches have markedly expanded the chemical toolkit available for studying protein synthesis and dynamics in living systems. By enabling the selective introduction of bioorthogonal chemical handles either at defined sites or across the proteome, these complementary strategies support diverse downstream applications, including enrichment, imaging, and proteomic analysis. Collectively, they provide a versatile foundation for investigating complex biological processes in specialized contexts such as neurobiology, where precise and dynamic protein labeling is essential for mechanistic insight.

## 3 Bioorthogonal Click Chemistry for Fluorescence Microscopy Studies in Neurobiology

Fluorescence microscopy techniques are central to cellular neurobiology, enabling the visualization of protein localization and interactions in both fixed and living cells. Alterations in protein processing, turnover, and transport are associated with numerous neurological disorders, making the ability to track these changes particularly important. Conventional live‐cell fluorescence microscopy typically relies on large fluorescent proteins for tagging. Although highly versatile, these bulky tags can, in some cases, alter the function, localization, or trafficking of the target protein. The development of super‐resolution microscopy (SRM) techniques such as stimulated emission depletion (STED), stochastic optical reconstruction microscopy (STORM), and photoactivated localization microscopy (PALM) has further increased the demand for labeling approaches that position fluorophores as close as possible to the protein of interest. Efforts to reduce linkage error by using smaller binders, such as nanobodies, affimers, and aptamers, have provided improvements [35, 36, 37], yet direct labeling of proteins with small organic dyes in living cells remains technically challenging.

Several self‐labeling protein tags based on engineered enzymes have been developed for this purpose, including HaloTag [38], SNAP‐tag [39], and CLIP‐tag [40], which enable covalent attachment of fluorescent dyes to a protein of interest using tag‐specific small‐molecule ligands (i.e., benzylguanine‐, benzylcytosine‐, or haloalkane‐based substrates for SNAP‐tag, CLIP‐tag, and HaloTag, respectively). Importantly, these tags can be used orthogonally, enabling robust and flexible multiplex labeling [40]. However, because these approaches rely on relatively large enzymes (derived from alkyltransferases or haloalkane dehalogenases; ≈20–35 kDa) and require expression of fusion proteins, they share many of the same limitations as conventional fluorescent proteins, including potential interference with protein folding, function, or localization. GCE with UAAs, combined with bioorthogonal click chemistry, has therefore emerged as a powerful method for incorporating small organic dyes directly into proteins at defined sites, enabling minimally invasive labeling and high‐precision fluorescence microscopy [26, 27, 28, 29, 30].

For such studies, stop codon suppression is used to introduce the UAA carrying the reactive handle site‐selectively into the protein of interest. Both the SPAAC and SPIEDAC reactions have been applied for bioorthogonal protein labeling in cells. However, SPIEDAC ligations are particularly relevant for fluorescence microscopy applications as tetrazine–fluorophore conjugates can undergo fluorogenic reactions with genetically encoded UAAs, thereby reducing background from unreacted dye [41, 42]. In addition, SPAAC labeling can be kinetically limiting in live‐cell experiments [30, 43], whereas tetrazine ligations generally proceed with substantially faster reaction rates, especially when highly reactive strained alkenes or alkynes, such as TCO and BCN derivatives, are used (Figure 3). Although exact reaction kinetics in genetically encoded systems depend on the specific UAA, tetrazine substitution pattern, and experimental environment, direct quantitative comparison of reported rate constants across studies is further complicated by variations in experimental design, including differences in solvent systems and measurement techniques. Nevertheless, under defined conditions, second‐order rate constants spanning 10^2^–10^5^ M^−1^ s^−1^ have been measured for tetrazine ligations of genetically encoded strained UAAs in a protein context [44]. Overall, SPIEDAC ligations proceed at rates that are well suited for efficient protein labeling under live‐cell conditions, contributing to their widespread use in fluorescence microscopy applications.

The suitability of SPIEDAC labeling for fluorescence microscopy was initially demonstrated in conventional cell lines, in experiments using epidermal growth factor expressed in mammalian cells with BCN‐ and TCO‐containing amino acids, followed by a rapid fluorogenic labeling reaction and conventional fluorescence microscopy [26]. SRM techniques were later implemented in studies of the insulin receptor and the influenza virus hemagglutinin protein [30]. In this study, it was also demonstrated that two different UAAs with orthogonal reactivity could be incorporated in response to the same codon using the same aaRS/tRNA pair in a pulse‐chase approach, in which the UAAs were added at different time points. This enabled downstream click reactions with different tetrazine‐functionalized fluorophores, allowing dual‐color imaging. This orthogonal pulse–chase labeling approach could enable dynamic processes such as protein sorting and transport to be studied more precisely in a time‐dependent manner using bioorthogonal click labeling. Beyond this, GCE was applied to other advanced imaging methods, including click‐enabled DNA‐PAINT. DNA‐PAINT [45] typically relies on antibody‐based labeling to place short DNA “docking strands” on target proteins. A complementary “imager strand,” carrying a fluorophore, is then added and transiently hybridizes with these docking strands, producing blinking events for super‐resolution imaging. In click‐PAINT [46], the docking strand carries a tetrazine handle and is clicked directly onto a UAA‐containing protein, after which the imager strands are applied as usual.

While some of these original studies focused on tuning the click reaction, others pursued further optimization of the translational machinery for GCE in mammalian cells. Such optimization can, in principle, be achieved at the level of the orthogonal tRNA, the components of the translational termination machinery, or the orthogonal tRNA synthetase. For example, engineered Pyl tRNAs [47], a mutant release factor eRF1 designed to reduce competition at the amber stop codon [48], and a PylRS variant fused to a nuclear export signal (NES) [46] have each been shown to enhance UAA incorporation, although these approaches were not systematically combined and evaluated across different mammalian cell types. Additional strategies and more recent advances in GCE efficiency, including extensive developments in the engineering of PylRS/tRNA^Pyl^ systems, have been extensively reviewed recently [33, 34] and will therefore not be covered here.

In the context of neurobiology, early labeling and microscopy studies were initially carried out in conventional cell lines rather than primary neurons. These include site‐specific labeling of NMDA receptors [49], bioorthogonal tagging of the Shaker B potassium channel [50], and SPIEDAC‐based labeling strategies used to monitor amyloid precursor protein (APP) processing [51], as also reviewed previously [52]. Clickable labeling has only recently been implemented in primary neurons, overcoming earlier challenges associated with studying these proteins in their native context and enabling their visualization with both conventional and SRM.

In one of the studies, SPIEDAC labeling was established to carry out advanced fluorescence microscopy studies of the neurofilament light chain (NFL) [53]. Using a variety of fluorophores, the authors visualized click‐labeled NFL by confocal and STED microscopy and also performed dual‐color labeling to distinguish different pulse–chase–labeled NFL populations. Furthermore, CRISPR/Cas9 genome editing was used to fluorescently label endogenous NFL by introducing a clickable UAA into the protein. This work represented the first demonstration of combining CRISPR/Cas9 genome editing with bioorthogonal click chemistry for fluorescence microscopy. However, because the UAA was incorporated as part of a C‐terminal tag rather than at an internal residue of the protein, the method did not fully exploit the site‐specific precision typically afforded by GCE‐based labeling. In addition, the overall efficiency of combining GCE with CRISPR/Cas9, particularly in primary neurons, remains low.

In another study, a SPIEDAC approach was used to label transmembrane AMPA receptor regulatory proteins in living neurons and organotypic brain slices [54]. A click labeling approach was particularly relevant in this case as cell‐impermeable tetrazine dyes were used to target masked epitopes on these proteins that could not be studied using traditional antibody‐based approaches. Transmembrane proteins are central to neuronal function, yet their epitopes are often masked or inaccessible, making conventional labeling difficult. Developing robust methods to visualize their localization and trafficking is, therefore, particularly valuable.

Subsequently, the methodology was optimized for even more complex targets, including large transmembrane proteins such as axon initial segment components [55]. In this work, the cell‐adhesion molecule NF186 (the 186 kDa isoform of neurofascin) and the 260 kDa voltage‐gated sodium channel Nav1.6 were visualized in neurons using both conventional and STORM microscopy following click reactions with fluorophores. As previously mentioned, the transfection efficiency in primary neurons is inherently limited, which can hinder GCE‐based labeling. To overcome this, the study used adeno‐associated virus (AAV) vectors to more efficiently deliver the GCE machinery. AAV‐based transduction was demonstrated to improve GCE and click chemistry‐based fluorescent labeling of these complex neuronal proteins, laying the groundwork for other studies in primary neurons and living mice.

In addition to labeling individual proteins, click chemistry can be used to visualize newly synthesized proteins globally by fluorescence microscopy. FUNCAT, like the related proteomics‐based BONCAT approach, relies on the co‐translational incorporation of azide‐ or alkyne‐bearing amino acid analogs into newly synthesized proteins, such as the methionine analogs L‐azidohomoalanine (AHA) or L‐homopropargylglycine (HPG) [56, 57]. These analogs are structurally similar to methionine and are therefore incorporated into proteins by the cell’s endogenous translational machinery. In FUNCAT, the incorporated amino acid analog is subsequently conjugated to a fluorophore by CuAAC, enabling visualization of newly synthesized proteins by fluorescence microscopy. In neurons, sequential labeling with AHA and HPG has been used to visualize proteins synthesized in rat hippocampal neurons during two distinct temporal windows [57]. BONCAT, which is primarily used for proteomic analysis, is discussed below.

As introduced earlier, an alkyne‐containing puromycin analog can also be used to fluorescently label proteins [32]**.** This molecule enters the ribosomal A‐site as a tRNA mimic and becomes covalently attached to elongating nascent polypeptides, simultaneously terminating translation (Figure 5). The incorporated puromycin analog can then be detected or enriched through a click reaction. This strategy, first demonstrated using OPP, enables the visualization of sites of nascent protein synthesis via CuAAC with fluorescent azides. To gain spatiotemporal control, photocaged puromycin analogs were subsequently developed, allowing light‐triggered labeling of newly synthesized proteins in living neurons [58]. More recently, cell‐type‐specific labeling in *Drosophila* brains was achieved using a caged OPP analog (PhAc‐OPP) that is enzymatically activated by penicillin G acylase, allowing visualization and enrichment of newly synthesized proteins specifically in targeted neuronal populations [59].

## 4 Bioorthogonal Click Chemistry for Proteomic Studies in Neurobiology

The site selectivity afforded by stop codon suppression for UAA incorporation is extremely valuable for imaging individual proteins with high precision. However, some studies require insights into proteome‐wide changes occurring within the cells under investigation. These broader questions are best addressed using quantitative proteomic approaches, which provide comparative system‐level information about physiological differences between samples. Quantitative proteomic methods have been used to detect changes in protein populations in immortalized neuronal cell lines, primary cultured neurons, and brain tissue [60, 61, 62].

Conventional proteomics studies, however, typically rely on traditional label‐free quantification or label‐based methods, including stable isotope labeling by amino acids in cell culture (SILAC), isobaric tags for relative and absolute quantification (iTRAQ), and tandem mass tags (TMT). Although highly effective in vitro, these strategies either do not offer cell specificity or are not compatible with experiments in intact organisms, which limits their utility in complex biological systems. Cell specificity is often addressed through cell sorting, such as fluorescence‐activated cell sorting (FACS) and magnetic‐activated cell sorting (MACS), but such procedures can shear the long processes of neurons and glial cells, making many neuronal cell types incompatible with downstream proteomic analysis. Bioorthogonal labeling approaches that rely on the incorporation of UAAs provide a powerful alternative for proteome‐wide profiling and can be adapted for either bulk or cell‐type‐resolved analysis.

The previously mentioned BONCAT labeling strategy is one such example [56]. In its original form, BONCAT uses the cell’s endogenous translation machinery, meaning that all cells will incorporate methionine analogs, such as AHA and HPG, during protein synthesis (Figure 4). As in FUNCAT, a downstream click reaction is performed; however, in the case of BONCAT, a functionalized biotin molecule is used instead of a fluorophore. The resulting biotinylated proteins can then be selectively enriched using well‐established pull‐down strategies.

Early application of the BONCAT methodology included experiments in primary hippocampal neurons [57]. Subsequently, more in‐depth analysis was carried out in a study on the dopaminergic modulation of the hippocampal neuropil proteome [63]. After treating intact hippocampal neuropil sections with D1/D5 receptor agonists, more than 300 newly synthesized proteins were identified. Additional BONCAT studies in cultured hippocampal neurons were performed to investigate synaptic processes [64, 65] and in cortical glutamatergic neurons to monitor activity‐induced changes and seizure‐evoked protein synthesis [66].

Although BONCAT was successfully established in vitro, its extension to in vivo systems was not straightforward. Efficient incorporation of methionine analogs by the native translational machinery was expected to be limited in more complex models, including intact tissues, where endogenous methionine levels, restricted amino acid uptake, and more variable protein turnover could all reduce labeling efficiency. Nevertheless, early studies extended BONCAT to in vivo applications, enabling profiling of newly synthesized proteins in the *Xenopus* brain [67, 68], the rat optic nerve using intravitreal injections of AHA [69], and even in whole mammals using pulsed AHA feeding in mice, followed by quantitative analysis of multiple tissues, including the mouse brain [70]. Further applications included investigation of acute living brain slices derived from a mouse model of Alzheimer’s disease [71] and in vivo mouse neurodegeneration studies to analyze the proteome in a model of tauopathy [72]. The neuronal membrane proteasome was specifically probed in *Xenopus* tadpoles using in vivo BONCAT, where the authors optimized the labeling protocol to capture newly synthesized proteins within an exceptionally short window of approximately 30 min [73]. Another useful development in vitro is the combination of BONCAT with SILAC (BONLAC) labeling for quantitative proteomics. This approach was initially optimized in HEK293 cells before being translated to hippocampal slices and cultures [74, 75, 76].

Although these studies showed that BONCAT labeling can be applied in intact animals and for advanced studies, its lack of cell specificity limited its applicability to more complex biological questions. This limitation was overcome by the development of genetically encodable BONCAT, which restricts labeling to defined cell populations.

A key advance in this direction was the engineering of a mutant methionyl‐tRNA synthetase (MetRS), which selectively charges the methionine analog azidonorleucine (ANL), which is not recognized by the endogenous translational machinery [77]. First applied in cultured cells using an engineered E. coli MetRS [77], this approach was subsequently adapted for use in live mammals using the mouse MetRS^L274G^ variant. In pioneering work in mice using a conditional Cre‐loxP expression system [78], newly synthesized proteins in hippocampal excitatory and cerebellar inhibitory neurons were selectively labeled and enriched through cell‐specific expression of the mutant MetRS^L274G^ in a transgenic mouse line. Another advantage is that ANL‐BONCAT can be performed without global methionine depletion as ANL is selectively charged by the engineered MetRS, whereas methionine depletion is commonly used to enhance AHA incorporation in classical BONCAT.

This has positioned ANL‐based labeling as a powerful tool in neurobiology. Among diverse applications, this approach was used to metabolically label nascent neuronal proteins in an acute mouse model of multiple sclerosis, enabling selective enrichment and mass‐spectrometric profiling of the newly synthesized neuronal proteome during neuroinflammation [79]. It has also been applied to identify proteome changes associated with memory formation [80]. Furthermore, the development of an astrocyte‐specific MetRS^L274G^ mouse model enabled in vivo metabolic labeling of newly synthesized astrocyte proteins, revealing both shared and region‐specific astrocyte proteomic signatures across brain regions [81].

Although ANL‐BONCAT enables cell‐type‐specific labeling of newly synthesized proteins, it is still subject to features inherent to methionine‐based analog incorporation, including competition with endogenous methionine, which can influence labeling efficiency depending on methionine availability, restriction of incorporation to methionine residues, and the limited chemical diversity of available methionine analogs.

An alternative approach is THRONCAT, which uses the threonine analog β‐ethynylserine. This analog is efficiently accepted by endogenous threonyl‐tRNA synthetase (ThrRS) even in the presence of normal threonine, enabling robust labeling without amino acid depletion or genetic engineering [82]. However, because THRONCAT relies on the native translational machinery, UAA incorporation itself is not cell‐type‐specific. Nevertheless, the original study demonstrated its broad applicability by using THRONCAT in bacteria, mammalian cell lines, and even a *Drosophila melanogaster* model of Charcot–Marie–Tooth peripheral neuropathy, in which it was used to visualize and quantify protein synthesis in GFP‐positive motor neurons in vivo [82].

As introduced above, SORT enables proteome‐wide UAA incorporation through stochastic reassignment of sense codons using an orthogonal aaRS/tRNA pair [31]. In the initial description of the method [31], incorporation frequencies depended on the targeted codon, with the UAA inserted in mammalian cells at roughly 0.02–0.2% of decoding events relative to the native amino acid, as estimated by the authors. This orthogonal incorporation mechanism also allows SORT to be performed in complete media without amino acid depletion [31].

The original SORT study [31] also introduced a smaller cyclopropene (CP)‐lysine derivative to replace bulkier BCN‐ and TCO‐containing amino acids. While BCN and TCO support highly efficient bioorthogonal reactions, their larger steric footprint was expected to pose challenges when incorporated proteome‐wide—potentially multiple times within a single protein—motivating the use of the more compact CP‐containing UAA. The authors further demonstrated cell‐specific SORT labeling in *D. melanogaster*, generating flies that incorporated the UAA into newly synthesized proteins in ovarian germ cells in response to several sense codons [31].

The cyclopropene‐based SORT strategy was subsequently extended with the development of SORT with enrichment (SORT‐E), which enabled selective enrichment and mass‐spectrometric identification of SORT‐labeled proteins using a cleavable tetrazine–biotin probe [83]. Despite the substoichiometric incorporation characteristic of SORT, efficient enrichment was achieved. Moreover, targeting different sense codons enriched overlapping but distinct subsets of the proteome, while combining orthogonal tRNAs targeting multiple sense codons enhanced proteome labeling and increased proteome coverage [83].

In a subsequent study [84], SORT was extended to dissociated cortical cultures, organotypic brain slices, and the brains of live mice using viral transduction. Proteomic analysis of genetically targeted striatal neurons identified 1789 proteins, with enrichment of established neuronal and medium spiny neuron markers [84]. For in vivo labeling, the SORT machinery was delivered to the mouse brain using a stereotactically injected AAV vector, with PylRS expression driven by the neuron‐specific hSyn1 promoter and the orthogonal tRNA expressed from a U6 promoter [84].

## 5 Current Challenges and Outlook

Bioorthogonal labeling strategies offer exceptional selectivity and minimal biological perturbation, enabling precise protein labeling in a range of neurobiological systems, from cell culture models to intact neural tissue. These approaches enable high‐resolution imaging at single‐amino acid precision while also supporting robust proteomic analyses. Bioorthogonal proteome labeling allows the capture of newly synthesized (nascent) proteins within defined temporal windows and, with engineered aaRSs, facilitates cell‐type‐specific proteomic profiling. Collectively, these advances have expanded the repertoire of experimental tools in neurobiology, allowing molecular analyses with a level of precision that is difficult to achieve using traditional methods.

Nevertheless, several challenges remain that limit the full potential of these approaches. First, genetically encoded UAA‐incorporation systems typically rely on transgenic or viral delivery of engineered aaRSs, yet these approaches are not uniformly efficient across cell types and can exhibit variable expression in complex neural systems. Second, UAAs must also be delivered and incorporated at sufficient concentrations without inducing metabolic burden or toxicity; however, their uptake, stability, and bioavailability in vivo continue to pose challenges. Third, selecting an appropriate click chemistry reaction requires balancing reaction kinetics, biocompatibility, and probe size, all of which influence the labeling efficiency and the potential perturbation of proteins or cellular physiology.

### 5.1 Fluorescence Microscopy

From a microscopist’s perspective, UAA‐based strategies represent one of the most powerful approaches for minimally invasive protein labeling, as they enable site‐specific incorporation of bioorthogonal handles that can be conjugated to fluorophores via click chemistry. This substantially reduces linkage or labeling errors, an especially critical advantage for SRM, where even nanometer‐scale offsets between the fluorophore and the target epitope can distort reconstructed structures [85, 86]. The minimal size of both the bioorthogonal handle and the resulting fluorophore also helps reduce steric perturbation of the labeled protein, lowering the risk of interfering with native protein function or cellular physiology.

These considerations are particularly important for neuronal proteins, which often reside in crowded and spatially restricted subcellular domains, such as synapses, dendritic spines, the axon initial segment, and nodes of Ranvier, where bulkier tags (e.g., fluorescent proteins or antibodies) may hinder proper localization, alter mobility, or fail to access the target site. GCE overcomes these constraints by enabling installation of a minimal tag (UAA + click‐dye) at a precisely defined residue.

However, the broader application of GCE remains limited by several practical challenges. The full GCE machinery—including the orthogonal aaRS/tRNA pair—must be introduced into the host cell, a significant hurdle in primary neurons, which are notoriously difficult to transfect efficiently. As a consequence, the overall UAA incorporation can be low. In addition, the protein of interest must be engineered to contain an amber codon at the desired labeling position. The efficiency of amber suppression varies substantially depending on the surrounding sequence context [87]; thus, even the same protein expressed in the same cell type can show dramatically different incorporation efficiencies depending on the chosen site. Together, these factors mean that each new target protein often requires iterative optimization to identify suitable expression conditions and viable UAA incorporation sites.

One strategy to overcome these challenges is to begin with simpler cellular models, such as immortalized neuronal cell lines (e.g., ND7/23 or N1E‐115) or even HEK293T cells, which have been widely employed in prior click chemistry‐based studies of neuronal proteins [53, 54, 55]. Using these systems simplifies experimental workflows and substantially reduces optimization time, allowing efficient refinement of UAA incorporation and labeling conditions before transitioning to more demanding primary neuronal cultures or in vivo models.

Furthermore, the issue of low transfection efficiency can be addressed by the application of alternative methods, such as transduction with viral vectors [55, 84] and electroporation [54]. Another strategy is the use of stable cell lines, which eliminate the variability inherent to transient transfections. For example, PiggyBac transposition has been employed to generate embryonic stem cells, mouse embryonic fibroblasts, and human induced pluripotent stem cell (iPSC)–derived lines and organoids that stably express the GCE machinery [88, 89]. More recently, genomic integration of GCE machinery has enabled dual stop‐codon suppression in mammalian cells, extending this strategy to more complex and modular GCE configurations [90].

However, while these stable‐integration strategies are powerful, they are not universally applicable. In particular, approaches such as PiggyBac‐mediated genomic insertion are generally far less practical for postmitotic primary neurons, both because stable genomic modification is more difficult to establish in these cells and because generating neuron‐specific stable lines is inherently limited compared to dividing cell models. Moreover, each orthogonal aaRS/tRNA pair requires its own dedicated stable integration, which becomes increasingly challenging given that the tRNA expression level often remains limiting even after genomic insertion. For these reasons, viral delivery or prior optimization in precursor models (e.g., neuronal progenitors, iPSCs, or immortalized cell lines) remains a more feasible strategy when the final application involves mature neurons.

Unwanted readthrough of endogenous stop codons is another potential concern in GCE systems, although no study to date has reported an appreciable background arising from amber suppression under standard experimental conditions. Instead, the more considerable challenge is the strong competition between the suppressor tRNA and the endogenous translation termination machinery, which often limits the overall UAA incorporation efficiency. Several strategies have been explored to mitigate potential off‐target UAA incorporation or to reassign codons more selectively. These include designer membraneless organelles that compartmentalize the amber codon suppression machinery within the host cell [91, 92, 93] and quadruplet (4‐base) codon suppression systems [94]. Quadruplet decoding has recently been demonstrated in *Caenorhabditis elegans*, and the authors used it for the optogenetic control of Cre recombinase and to ablate specific neurons [95]. However, the overall incorporation efficiency remains lower than that achieved with standard stop codon suppression, and implementation in mammalian cells is still limited. In general, while these different advanced GCE systems are conceptually powerful, significant hurdles remain for their application in complex biological systems, and in particular in dissociated neurons and intact nervous tissue.

Finally, click chemistry‐based labeling of endogenous proteins within their native environment represents an important next step. Current examples rely on the overexpression of the target protein, potentially leading to artifacts. Recent progress toward endogenous click chemistry‐based labeling in neurons has been achieved using CRISPR/Cas9‐mediated knock‐in strategies [53]. In this study, a sequence encoding a short C‐terminal peptide tag containing a single amber codon was introduced into the endogenous *Nefl* locus in primary neurons, enabling the incorporation of a clickable UAA directly into the endogenously expressed protein. The tag was positioned at the C terminus to minimize disruption to NFL expression in cases where amber suppression was incomplete; however, in principle, such genome‐editing approaches could be used to introduce UAAs at other positions within endogenous proteins. Despite these advances, the strategy remains fundamentally constrained. Because amber suppression has limited efficiency, introducing a stop codon at the DNA level into an essential endogenous protein may result in a fraction of translation events terminating at the engineered stop codon, thereby producing truncated protein variants.

A complementary strategy involves endogenous labeling via RNA editing, where a recent study has shown that GCE can be combined with RNA editing to incorporate UAAs into endogenous proteins in conventional cell lines and neurons [96]. In this work, the authors reported the labeling of Nav1.6, Cav2.1, and NFL in primary neurons, although detailed information on the specific codon positions used for UAA incorporation was not fully provided for all of these proteins. In any case, mRNA‐editing–based approaches provide a powerful strategy for labeling native proteins directly from their endogenous transcripts; however, their broad applicability and robustness across different proteins and cell types remain to be demonstrated.

More recently, intracellular protein editing using split inteins has enabled site‐specific introduction of UAAs into endogenous proteins [97]. In this approach, a UAA‐containing donor is generated by GCE in *E. coli*, purified, and delivered into mammalian cells, where its cargo is incorporated into the target protein through tandem split intein‐mediated trans‐splicing. The introduced bioorthogonal handle can subsequently be conjugated to fluorophores or affinity tags, as demonstrated for endogenous calnexin. However, the approach still requires prior genome editing to introduce an intein acceptor sequence into the endogenous target, potentially limiting its applicability when such modification interferes with protein function.

Despite the remaining hurdles, all of these advances open new possibilities for high‐precision studies of neuronal proteins using both conventional and SRM. In particular, they enable labeling of proteins with masked epitopes, those embedded in crowded molecular environments, and proteins that are otherwise perturbed by bulkier labeling methods, including NF186, NaV1.6, NFL, GABA receptor subunits, and AMPA‐receptor auxiliary subunits such as TARP γ2/γ8 [53, 54, 55, 98]. Beyond these applications already demonstrated in neurons, bioorthogonal labeling strategies developed in conventional cell lines offer additional opportunities for future neurobiological studies. For example, bioorthogonal labeling has been used to develop genetically encoded FRET‐based sensors for metabotropic glutamate receptors [99, 100]. For these types of genetically encoded FRET sensors, bioorthogonal labeling is powerful because it enables site‐specific installation of donor and acceptor fluorophores that report event‐dependent proximity changes in fluorescence. Combining bioorthogonal labeling with enzymatic tagging enables even more advanced dual‐ and triple‐labeling strategies to monitor distinct protein conformations. Such approaches have been used to investigate previously unknown conformational changes in metabotropic glutamate receptors [99, 100]. Initially, a CuAAC‐based labeling strategy was combined with SNAP‐tag labeling [99], while a subsequent study introduced a third, site‐specific SPIEDAC‐based labeling reaction [100]. These approaches enabled the analysis of receptor dynamics in response to orthosteric and allosteric modulators and revealed the existence of an intermediate state between “open/resting” and “closed/active” conformations.

Bioorthogonal labeling has been used to develop approaches for visualizing proteolytic processing of APP and the generation of amyloid‐β (Aβ) [51, 101]. One of the studies described a dual labeling strategy for APP by combining a C‐terminal SNAP‐tag with UAA‐mediated site‐specific labeling of the Aβ region of APP, enabling simultaneous visualization of APP and its cleavage products in living cells with minimal perturbation [51]. Similarly, in the molecular beaker design study [101], GCE was used to introduce a clickable UAA into the Aβ region, while a HaloTag on the C‐terminus of APP was used to attach a fluorescence quencher. Proteolytic release of Aβ separates the fluorophore–quencher pair, generating a fluorescence signal that enables the visualization of Aβ production in living cells.

### 5.2 Proteomic Investigations

BONCAT, SORT, and puromycin‐based approaches such as OPP provide complementary strategies for probing proteome dynamics in neurons. Whereas BONCAT and SORT enable click chemistry‐based enrichment and mass spectrometry identification of newly synthesized proteins, OPP offers a rapid readout of active translation through direct labeling of nascent chains without requiring amino acid deprivation or genetic manipulation. However, because puromycin analogs induce premature translation termination and generate truncated products that are often rapidly degraded, OPP is generally better suited for measuring translational activity than for enrichment‐based proteomic analyses. On the other hand, both BONCAT and SORT offer powerful strategies for neuroproteomics by enabling click chemistry‐based enrichment of labeled proteins. Compared to microscopy studies that favor fully bioorthogonal copper‐free reactions, the reactions used for enrichment are frequently copper‐catalyzed. However, since they are performed on lysates, copper toxicity, which is a concern for live‐cell labeling, is not a major concern in this context.

BONCAT provides metabolic labeling of newly synthesized proteins without requiring codon reassignment and can be performed using either the endogenous MetRS or engineered MetRS variants. This flexibility makes it suitable for both bulk and cell‐type‐specific analyses in vivo, with engineered MetRS variants offering markedly improved selectivity and efficiency. However, even with engineered MetRS, BONCAT remains constrained by competition with endogenous methionine, which can limit the labeling efficiency.

Recent developments have begun to address these limitations. A next‐generation transgenic mouse line carrying multiple copies of the mutant MetRS (3×MetRS*) greatly increases ANL incorporation in targeted cell types, enabling highly sensitive, cell‐type‐specific proteomic profiling in vivo without the need for methionine depletion [102]. In addition, the temporal resolution of BONCAT has been improved by the development of a light‐activatable ANL analog (Opto‐ANL) [103]. Opto‐ANL can be loaded into neurons and subsequently activated using mild UV light, triggering the rapid release and incorporation of ANL in cells expressing MetRS^L274G^. This strategy produces faster and more robust labeling than ANL itself and allows the detection of dynamic proteomic changes, such as insulin‐induced increases in protein synthesis. Together, these innovations expand the spatial and temporal precision of BONCAT, offering new opportunities for cell‐type‐specific and temporally resolved neuroproteomic studies.

Another concern related to MetRS‐based approaches is that BONCAT depends on the presence of endogenous methionine residues. Although nascent proteins begin with an N‐terminal methionine, this residue is frequently removed cotranslationally under physiological conditions, reducing the number of proteins that can be labeled at their N‐terminus. Moreover, methionine is a relatively low‐abundance amino acid, so overall proteome coverage can be limited by the scarcity of internal methionine residues available for substitution. As introduced earlier, THRONCAT provides an alternative approach that can be performed in complete media; however, its applications remain limited, and its reliance on the endogenous translational machinery precludes cell‐type‐specific proteome labeling. More recently, engineered TyrRS and TrpRS variants have been developed that enable rapid orthogonal incorporation of distinct amino acid analogs, allowing multiplexed BONCAT labeling in mixed bacterial populations [104]; however, this labeling approach has so far been demonstrated only in bacteria and has not yet been extended to mammalian systems.

Unlike analog‐based metabolic labeling, which relies on endogenous tRNAs and therefore restricts incorporation to the canonical codons of the substituted amino acid, SORT allows deliberate codon selection, providing greater control over the residues targeted across the proteome compared with BONCAT. However, SORT requires the delivery and expression of both an orthogonal tRNA and its corresponding aaRS, increasing the experimental complexity compared with metabolic labeling approaches such as BONCAT, particularly for in vivo applications. This additional complexity may have contributed to its relatively limited application in neurobiology. In addition to this experimental complexity, UAA incorporation by SORT occurs at relatively low frequencies at individual targeted sense codons. Nevertheless, the widespread occurrence of these sense codons across the proteome, together with the specificity of bioorthogonal enrichment strategies, enables robust proteomic detection. The effects of low incorporation frequencies can be further mitigated by targeting multiple sense codons, thereby increasing proteome coverage [83].

Another drawback of both BONCAT and SORT is that because they selectively label newly synthesized proteins, they may be less effective in cells with low protein turnover and can miss changes in stable, long‐lived protein populations. For example, if a highly stable protein is present before amino acid labeling and is subsequently downregulated in response to a treatment or physiological perturbation, neither BONCAT nor SORT will detect this decrease. In such cases, enzymatic proximity‐labeling approaches such as BioID, engineered ascorbate peroxidase (APEX), or its improved variant APEX2, and TurboID may be more suitable [105]. Their principal advantage is that labeling is independent of nascent protein synthesis, enabling the analysis of pre‐existing proteins. Bioorthogonal chemistry has also been integrated with proximity labeling; for example, the alkyne‐containing APEX2 substrate propargyl tyramide has been applied in acute mouse brain slices, where subsequent click conjugation to an azide‐functionalized fluorophore enabled visualization of APEX2‐mediated labeling [106]. Comprehensive overviews of the use of these labeling approaches in neural systems have been reported elsewhere [105, 107, 108], and therefore, we do not discuss them in detail here.

More recently, a multiscale photocatalytic proximity‐labeling platform termed MultiMap has been introduced [109]. This method employs the visible‐light photocatalyst eosin Y (EY), which can be conjugated to an antibody targeting a protein of interest. In this study, EY was coupled to cetuximab to target the epidermal growth factor receptor (EGFR) at the cell surface. Upon light irradiation, EY catalytically activates multiple classes of photoreactive biotin probes, including diazirine‐, aryl‐azide‐, phenol‐, and hydrazide‐based probes, resulting in biotinylation of EGFR and nearby proteins over tunable distances ranging from ~100 to 3000 Å. Earlier proximity‐labeling approaches, including enzyme‐based APEX and BioID or photocatalytic systems such as μMap, typically operated within a single, fixed labeling radius defined by the reactive species. In contrast, MultiMap integrates multiple probes within one platform, enabling multiscale mapping of protein environments without switching techniques. Although not yet applied in neurons, it was successfully used to profile cell–cell synapses induced by bispecific T cell engagers and chimeric antigen receptor T cells. Given its tunable spatial resolution, this strategy may also be of interest for investigating complex protein organization at neuronal synapses.

Looking ahead, combining or multiplexing proximity‐ and photocatalytic labeling strategies with BONCAT‐ or SORT‐based methods for nascent proteome labeling may provide a more comprehensive view of proteomic and interactomic changes in neural systems.

## Funding

This work was supported by the Deutsche Forschungsgemeinschaft (Grant 317530061) and HORIZON EUROPE Marie Sklodowska‐Curie Actions (Grant 101068378).

## Conflicts of Interest

The authors declare no conflicts of interest.
